# Under the Sea: Detection of Explosives Underwater Using Raman Spectroscopy

**DOI:** 10.3390/molecules31172994

**Published:** 2026-08-27

**Authors:** Dominika Łucja Sobczuk, Karol Zalewski, Mateusz Szala, Grzegorz Siedlewicz, Vasile Sorin Balan

**Affiliations:** 1 Department of Explosives, Military University of Technology, 2 Kaliskiego Str., 00-908 Warsaw, Poland; 2 Institute of Oceanology of Polish Academy of Sciences, Powstańców Warszawy 55, 81-712 Sopot, Poland; 3 National Institute for Research and Development on Marine Geology and Geo-Ecology—GeoEcoMar, Mamaia Boulevard, No. 304, 900581 Constantza, Romania; sbalan@geoecomar.ro

**Keywords:** dumpsites, Raman spectroscopy, handheld analyzer, remote detection, water pollution

## Abstract

Secondary explosives such as 1,3,5-trinitro-1,3,5-triazacyclohexane (RDX) and 2,4,6-trinitrotoluene (TNT) were commonly used in the production of many types of munitions during World War II and still continue to be used, for example, 1,3,5,7-tetranitro-1,3,5,7-tetrazocane (HMX) and pentaerythritol tetranitrate (PETN). After the war, numerous remaining munitions were disposed of by dumping them into the Baltic Sea. The same was true for the Black Sea, but due to a lack of documentation about these dumpsites, the awareness of hazards and knowledge about the scale of the problem have significantly increased in the last 30 years. After decades spent in salty water, the shells of said projectiles corroded, and currently, they pose a risk of environmental contamination and explosion. Because of this, new underwater detection methods that are reliable, quick, remote and, at the same time, insensitive to the external interference of sea water are needed. The detection of explosives used in projectiles can be performed using field Raman spectrometers. Raman spectroscopy is an analytical method that is non-invasive: it allows one to perform analysis without taking samples out of the projectile. Field detectors are water-resistant due to the frequent need for decontamination. Therefore, in this study, a ThermoScientific Gemini analyzer (Waltham, MA, USA) was used. The purpose of the study was to determine how the Raman spectra of pressed explosives are influenced by two main factors: the distance between the Raman probe and the sample and the type of medium that stands between the Raman probe and the sample. For the purpose of this study, four explosives: TNT, RDX, PETN, and HMX were tested through various layers of distilled water, tap water, silica dispersion, Black Sea water and, finally, Baltic Sea water.

## 1. Introduction

**Figure 1:**
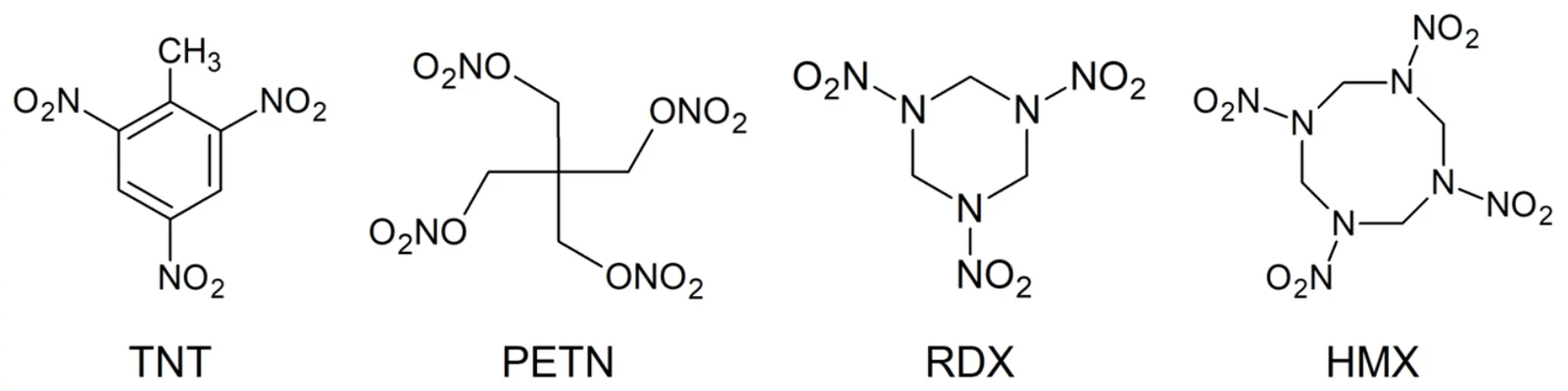
Chemical structure of secondary explosives: 2,4,6-trinitrotoluene (TNT), pentaerythritol tetranitrate (PETN), 1,3,5-trinitro-1,3,5-triazacyclohexane (RDX) and 1,3,5,7-tetranitro-1,3,5,7-tetrazocane (HMX).

After World War II, when the international conflict faded, another problem appeared: what should be done with the remaining munitions? Due to the disarmament agreed in Potsdam in 1945, at least 40,000 tons of munitions were dumped in the coastal waters of the Baltic Sea. Later, in 1952–1965, an amount of 200–300,000 tons was added to that count [1,2]. The situation was handled completely differently on the Soviet side of the conflict: there was no agreed place for the dumpsite and the amount of munitions was not known, which has made the current monitoring situation a lot more complicated. In the last 30 years, scientists from countries bordering the Black Sea have collected data on its seabed looking for the remains of potentially hazardous objects. During said investigations, they found shipwrecks with numerous UXO-items and located corridors and sites with a high density of targets [3]. As time passed, shells of said munitions rusted and began to pose a threat due to the fillings from munitions being released right into the water [4,5]. The fillings of high explosive munitions mostly consist of TNT, RDX or a TNT-HND mixture called hexanite. TNT and its metabolites are organic compounds that have toxic effects on living creatures, in this case marine organisms, when they accumulate in their tissue [6,7,8,9]. Many scientists have investigated this pollution problem by taking samples of water, sediments from potentially polluted areas or fish tissues and then testing these samples for the presence of harmful substances. In a few publications, remote methods of investigation have been used to identify detectable concentrations of high explosives (usually TNT, RDX, PETN, HMX) in sea water, but the idea of underwater detection of explosives using spectroscopic methods is still not popular [10,11,12,13] (Figure 1).

Raman spectroscopy was selected to check the measurement capabilities of commonly used devices. This method offers unquestionable advantages: non-invasiveness, high specificity and, most important in this case, not interfering with water, which makes Raman spectroscopy a perfect candidate for underwater detection. The hand-held Raman spectrometers used by police officers, fire departments and the army in Poland combine these advantages with a convenient size and weight of a few kilograms, as well as water resistance up to 30 min.

## 2. Results

The tests for each sample started with distilled water. After that, tap water and silica dispersions were used. Silica dispersions were used in order to simulate the conditions at the bottom of the Baltic Sea. Additionally, the spectra were also collected without any liquid. Tests were also performed using water collected from the bottom of the Black Sea and the Baltic Sea.

### 2.1. High Explosives with Laboratory Water Samples

#### 2.1.1. Pressed TNT

**Figure 2:**
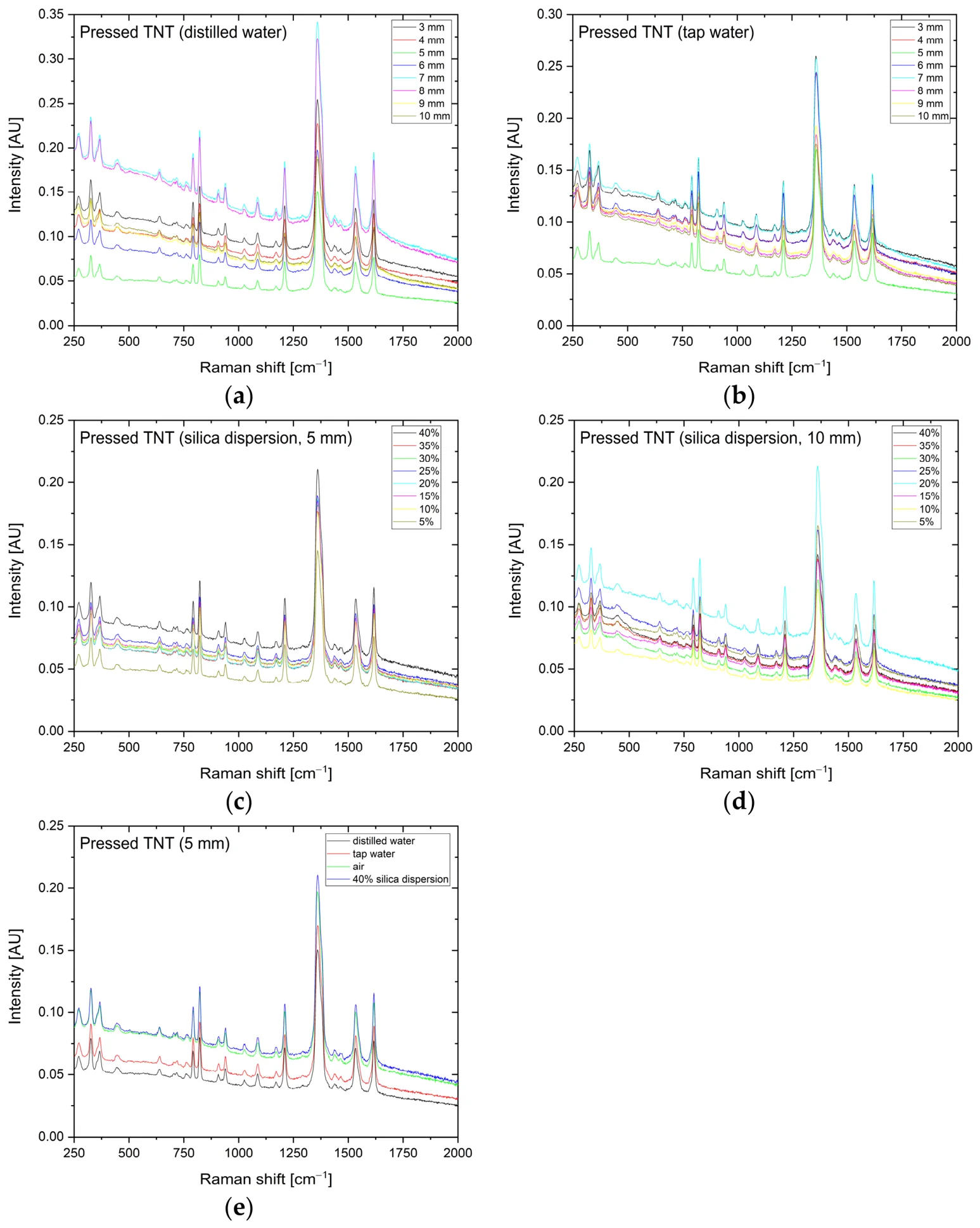
The Raman spectra of pressed TNT: (**a**) through distilled water, (**b**) through tap water, (**c**) through silica dispersions at 5 mm distance, (**d**) through silica dispersions at 10 mm distance, (**e**) through different media at 5 mm distance.

The influence of distilled water on the Raman spectrum of pressed TNT is presented on Figure 2a. The spectrum with the least intense baseline was registered at 5 mm distance, yet the characteristic bands are clearly distinguishable in all cases. Six main bands can be associated with TNT: 792 cm^−1^ (C-H out-of-plane bending), 822 cm^−1^ (-NO_2_ deformation), 1210 cm^−1^ (C_6_-C deformation), 1360 (-NO_2_ symmetric stretching), 1534 cm^−1^ (-NO_2_ asymmetric stretching) and 1616 cm^−1^ (C=C stretching) [14,15]. Similar results were achieved using tap water (Figure 2b). In both cases the strongest baseline intensity was observed when the distance was 7 mm. Gemini (software version: 1.10.0.7726) was able to successfully identify TNT at all distances both with distilled and tap water. Changing the medium had no significant influence on the spectra. The low intensity of the baseline at 5 mm distance was probably connected with the fact that this distance matched the focal length of the probe. Next, the spectra through silica dispersions at 5 and 10 mm distance (Figure 2c,d) were collected. A 5 mm distance was chosen because of both the focal point of the probe, which is approximately that value, and for giving the best outcome in previous scenarios. A 10 mm distance was chosen as the longest tested and potentially the most problematic due to the thickest layer of medium. The presence of the silica dispersions had no significant influence on the spectra both at 5 and 10 mm distance. All six main TNT bands are clearly distinguishable. Silica seems to influence the baseline intensity slightly, but with no predictable correlation. Gemini was able to successfully identify TNT through every dispersion. In Figure 2e the chosen spectra are compared with the spectrum through air. The presence of distilled water positively influenced the spectrum, lowering the baseline intensity in comparison to air. The spectra through 40% silica dispersion and air were almost identical.

#### 2.1.2. Pressed RDX/Wax Formulation

**Figure 3:**
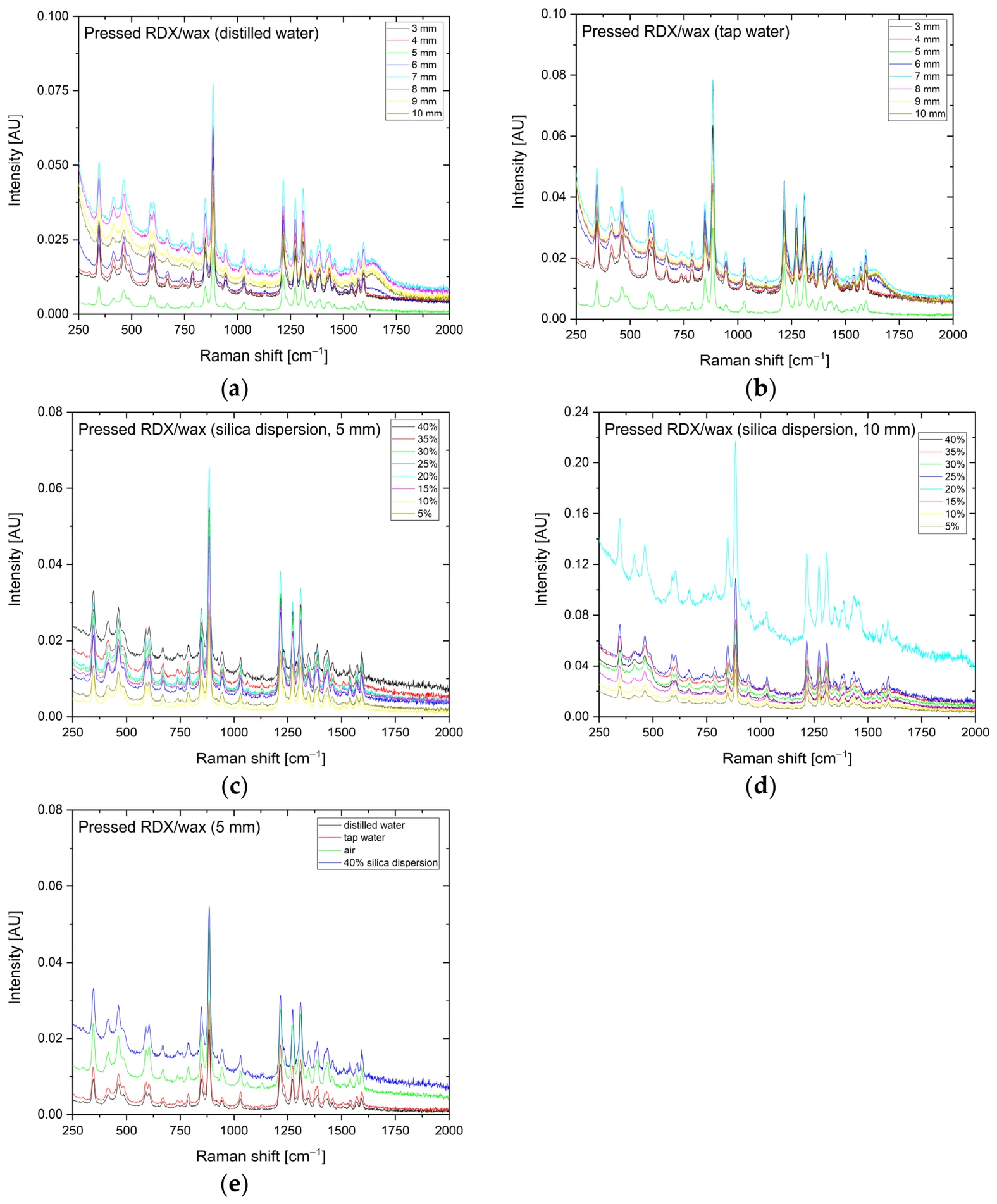
The Raman spectra of pressed RDX/wax: (**a**) through distilled water, (**b**) through tap water, (**c**) through silica dispersions at 5 mm distance, (**d**) through silica dispersions at 10 mm distance, (**e**) through different media at 5 mm distance.

The spectra of pressed RDX/wax formulation through distilled water are presented in Figure 3a. There are six easily detectable bands that can be used to identify RDX: 589 cm^−1^ (−NO_2_ wagging), 848, 883 and 1216 cm^−1^ (NC_2_ ring stretching), 1272 and 1310 cm^−1^ (−NO_2_ symmetric stretching) [14,16]. Tap water had no significant influence on the spectra (Figure 3b). In both cases the strongest baseline intensity was observed when the distance was 7 mm. Gemini was able to successfully identify RDX at all distances both with distilled and tap water. Again, the best results were achieved when the distance was 5 mm. The 40% silica dispersion led to the biggest increase in the baseline intensity at a 5 mm distance (Figure 3c). At a 10 mm distance the spectrum was significantly influenced by the 20% silica dispersion (Figure 3d). Nevertheless, characteristic bands were still clearly visible and Gemini was able to detect RDX in each case. In Figure 3e the chosen spectra are compared with the spectrum through air. Similarly to TNT, distilled and tap water positively influenced the spectra in comparison to air. Use of a 40% silica dispersion led to the biggest increase in the baseline intensity.

#### 2.1.3. Pressed HMX/Wax Formulation

**Figure 4:**
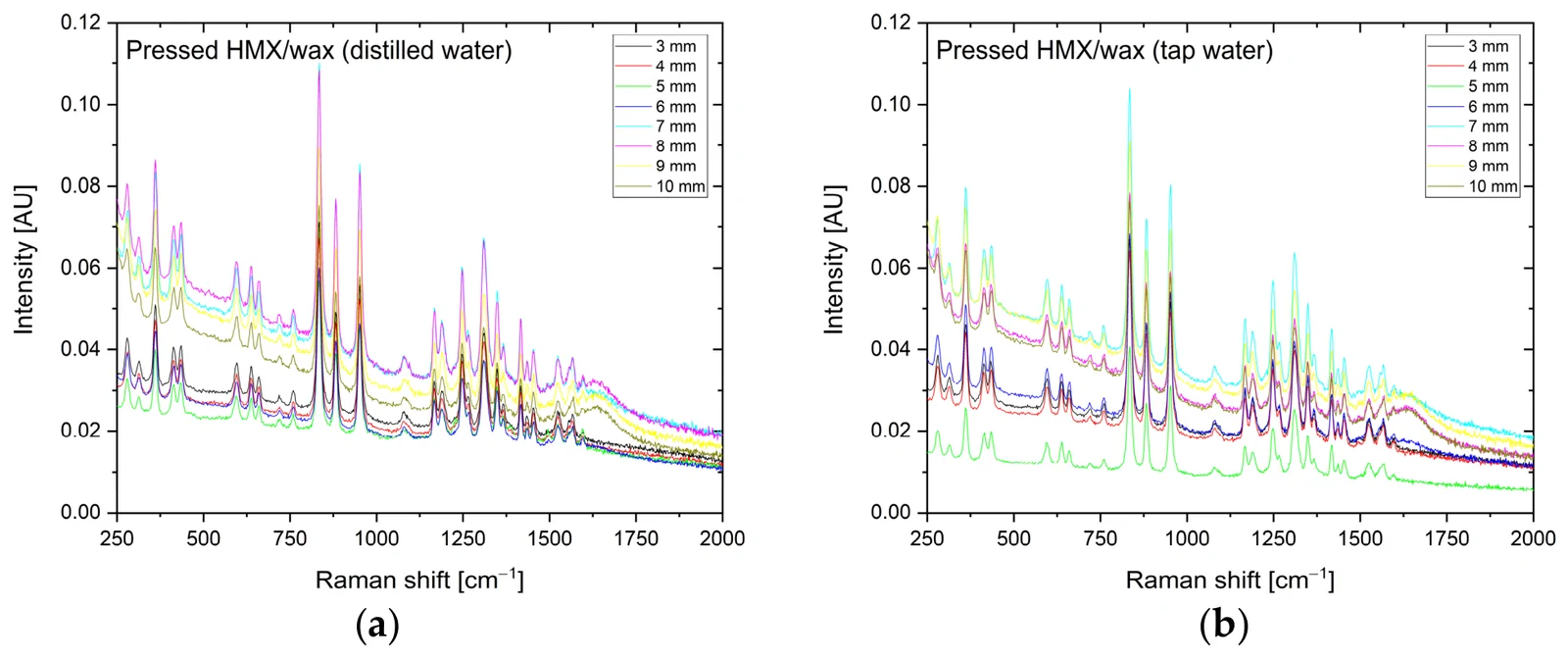

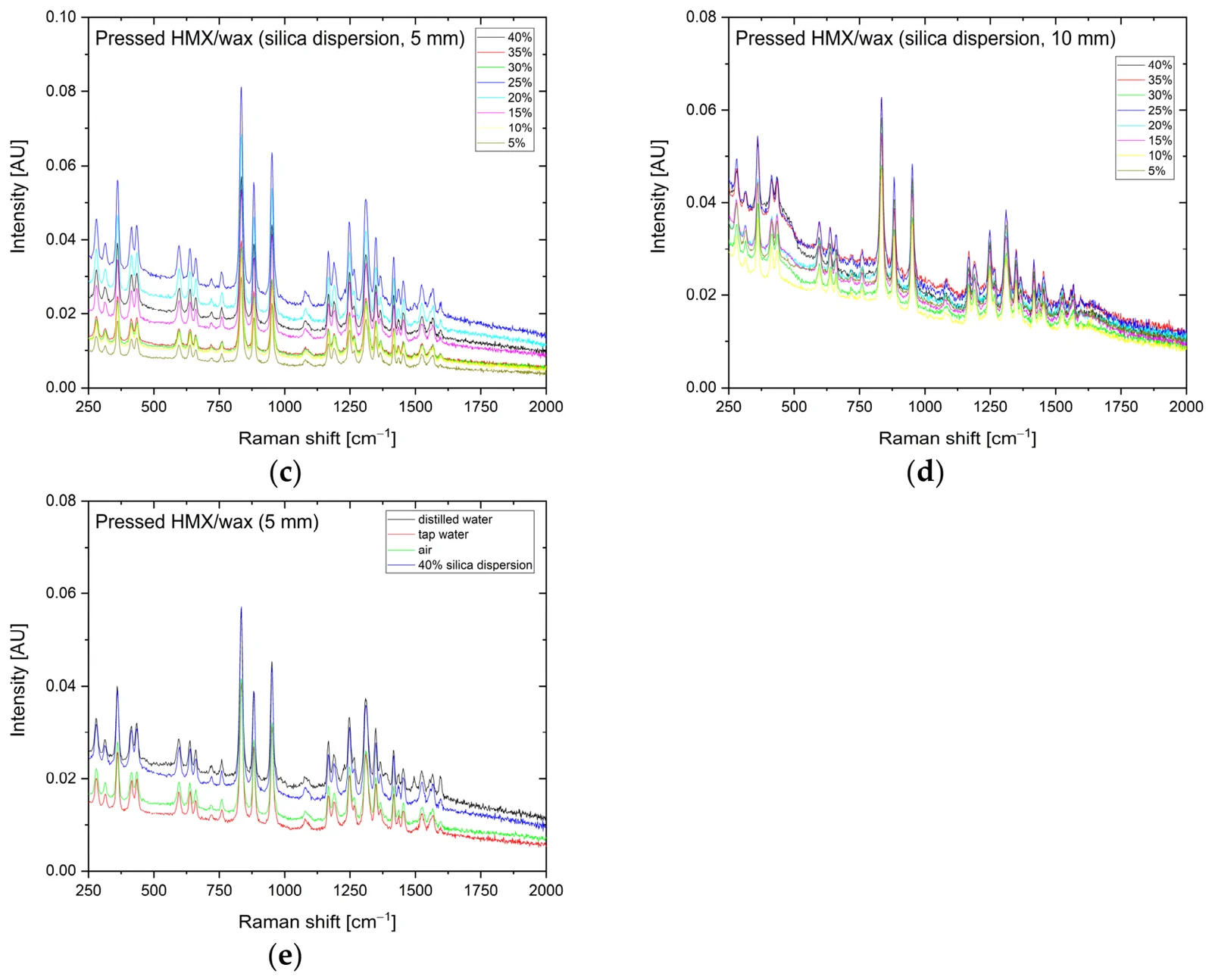
The Raman spectra of pressed HMX/wax: (**a**) through distilled water, (**b**) through tap water, (**c**) through silica dispersions at 5 mm distance, (**d**) through silica dispersions at 10 mm distance, (**e**) through different media at 5 mm distance.

The spectra of the pressed HMX/wax formulation through distilled water are presented in Figure 4a. Due to the very similar chemical structures of HMX and RDX, similar characteristic bands can be observed: 595 cm^−1^ (-NO_2_ wagging), 834, 881, 1168 and 1187 cm^−1^ (NC_2_ ring stretching), 951 cm^−1^ (N-N stretching), 1247 and 1309 cm^−1^ (-NO_2_ symmetric stretching) [14]. The spectra collected through distilled and tap water are similar (Figure 4b). The baseline intensity of the spectrum at 5 mm through tap water was even lower than that of distilled water. Gemini was able to successfully identify HMX at all distances both with distilled and tap water. Again, the best results were achieved when the distance was 5 mm. Spectra collected through the silica dispersions (Figure 4c,d) were inconclusive, similarly to the TNT and RDX results. There was no relation indicating that the increase in the content of silica in the dispersion led to an increase in the baseline intensity. At 5 mm distance the strongest baseline intensity was measured when a 25% silica dispersion was used. Unlike in the previous cases, the spectrum through distilled water had the most intense baseline in comparison to tap water, air and the 40% silica dispersion (Figure 4e).

#### 2.1.4. Pressed PETN/Wax Formulation

**Figure 5:**
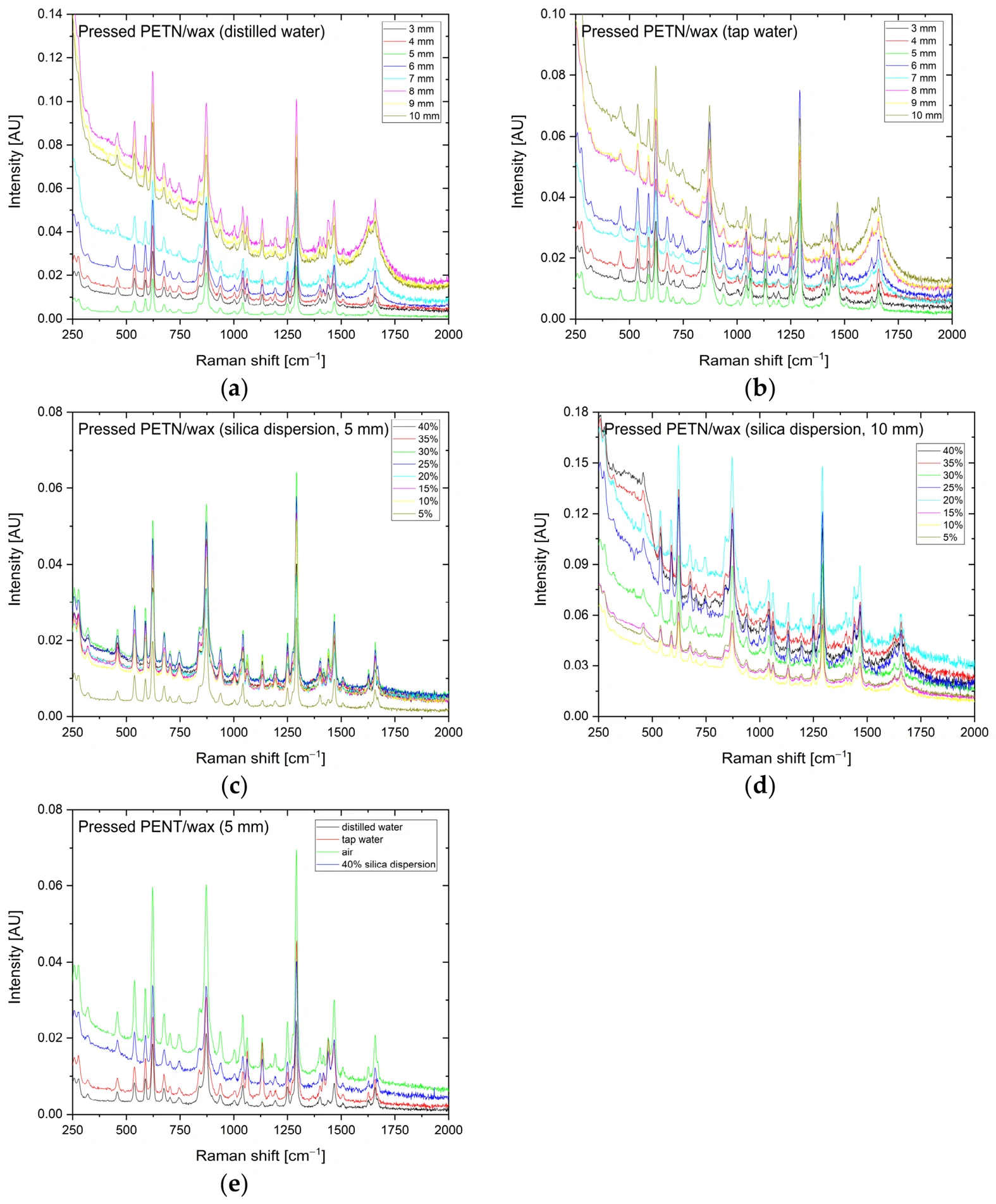
The Raman spectra of pressed PETN/wax: (**a**) through distilled water, (**b**) through tap water, (**c**) through silica dispersions at 5 mm distance, (**d**) through silica dispersions at 10 mm distance, (**e**) through different media at 5 mm distance.

The spectra of pressed PETN/wax formulations through distilled and tap water are presented in Figure 5a,b. Three strong characteristic bands remained distinguishable despite the intense baseline at greater distances. These bands are: 621 cm^−1^ (-NO_2_ in-plane deformation), 872 cm^−1^ (N-O stretching), and 1291 cm^−1^ (-NO_2_ symmetrical stretching) [14]. Despite the high content of wax (25%), Gemini managed to identify PETN in each case. Peaks with different intensities and widths appear in energetic materials in the Raman spectra in the region between 1500 and 1700 cm^−1^, as shown in Figure 5b. At 1490 cm^−1^ a peak associated with the scissor vibrations of the -CH2-group can be seen for PETN. At 1670 cm^−1^, an asymmetric stretching vibration of the nitro group can be seen. Increasing the thickness of the water layer between the sample and the detector causes the baseline to rise due to the amplification of the signal responsible for the scissor vibrations of the water molecule (1640 cm^−1^). The spectra collected through the silica dispersions (Figure 5c,d) were inconclusive like in previous cases. Nevertheless, PETN was positively identified. Similarly to TNT and RDX, both distilled and tap water influenced the spectra by lowering the baseline intensity in comparison to air (Figure 5e).

### 2.2. High Explosives with Environmental Water Samples

#### 2.2.1. The Black Sea

**Figure 6:**
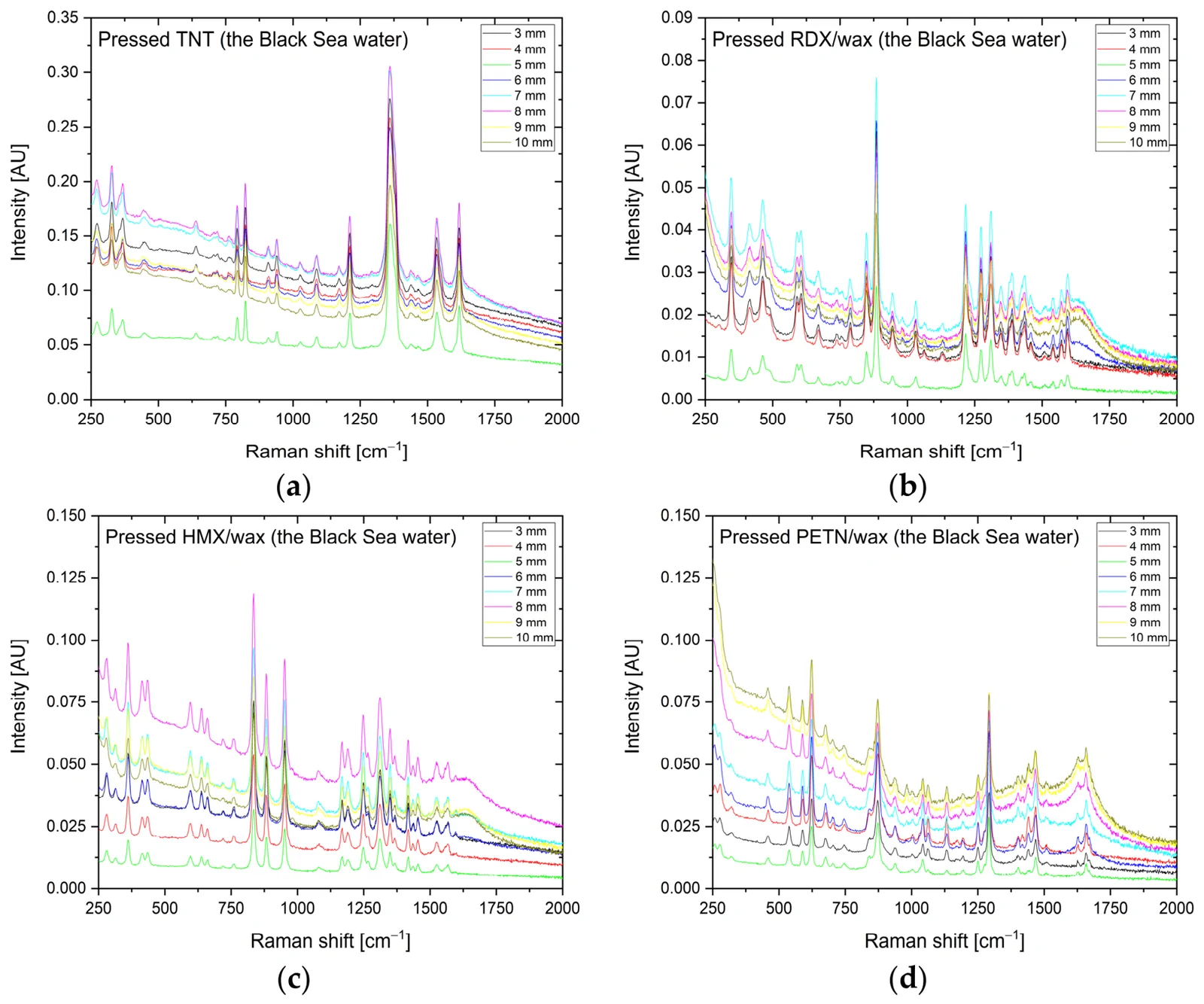
The Raman spectra of pressed charges of explosives through Black Sea water: (**a**) the Raman spectra of pressed TNT through Black Sea water, (**b**) the Raman spectra of pressed RDX/wax through Black Sea water, (**c**) the Raman spectra of pressed HMX/wax through Black Sea water, (**d**) the Raman spectra of pressed PETN/wax through Black Sea water.

The influence of Black Sea water on the Raman spectrum of the selected high explosives is presented in Figure 6a–d. The spectrum with the least intense baseline was registered at a 5 mm distance, yet the characteristic bands are clearly distinguishable in all cases. Gemini was able to successfully identify all high explosives at all distances.

**Figure 7:**
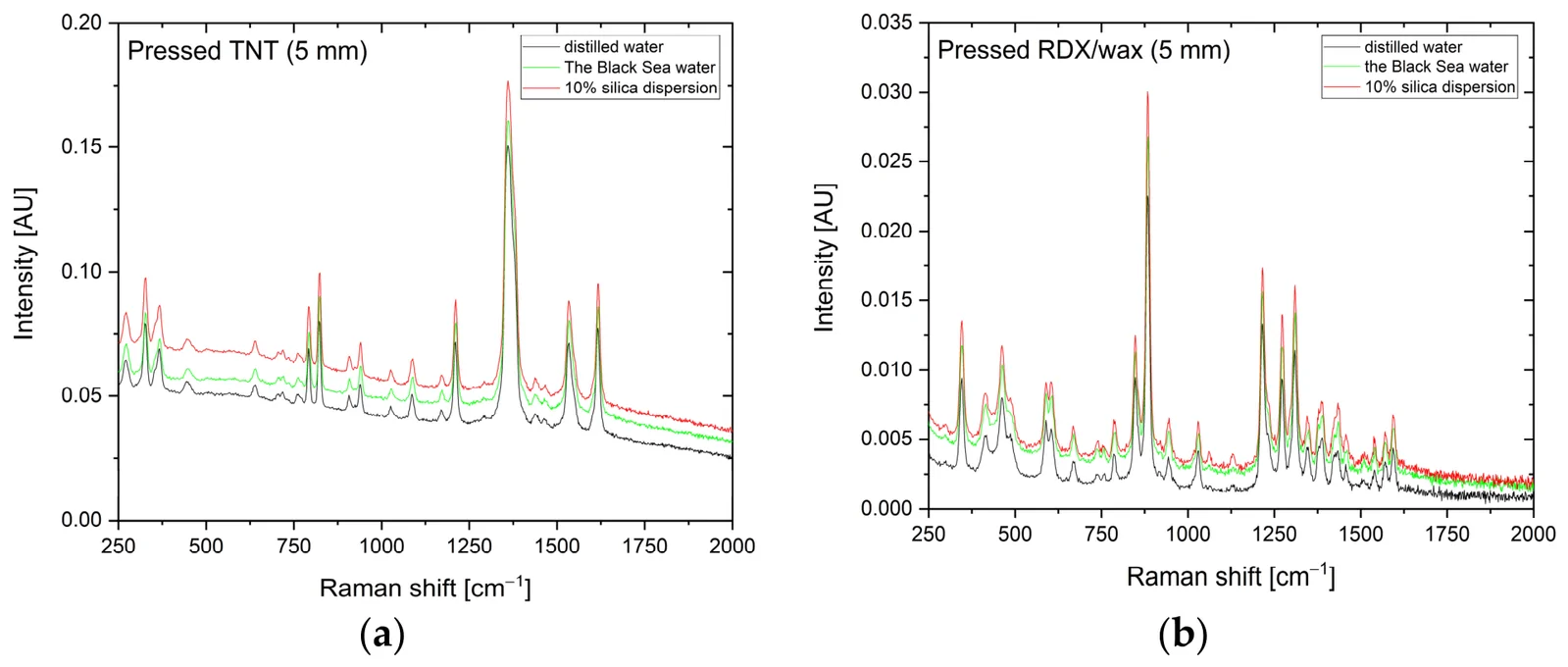

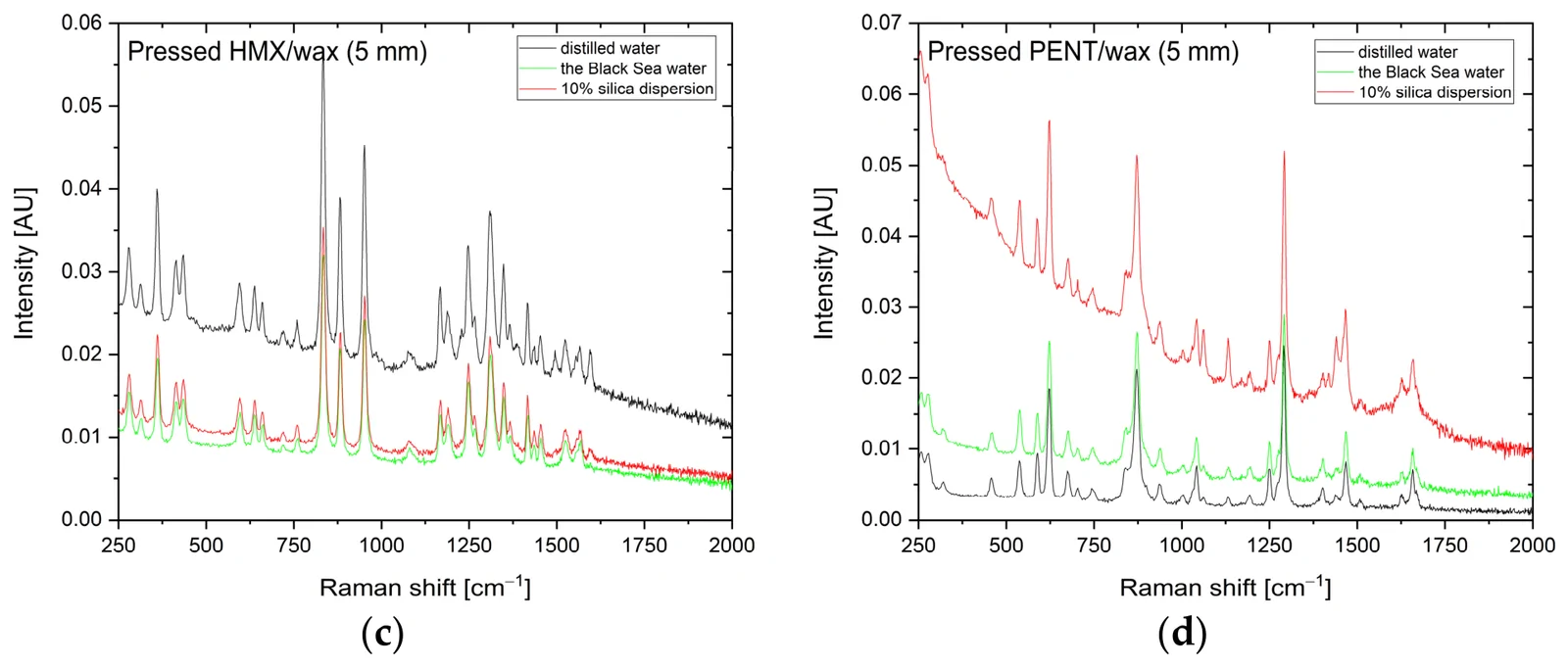
Comparison of the Raman spectra of pressed charges of explosives through Black Sea water, distilled water and 10% silica dispersion at 5 mm distance for: (**a**) TNT, (**b**) RDX/wax, (**c**) HMX/wax, (**d**) PETN/wax.

The spectra in Black Sea water are compared with distilled water and silica dispersion at 5 mm distance in Figure 7a–d. In the case of pressed TNT, the spectrum through Black Sea water lays in the middle between the distilled water and the 10% silica dispersion (Figure 7a). In the case of pressed RDX/wax, the spectra through Black Sea water and 10% silica dispersion are almost identical (Figure 7b). The same goes for the pressed HMX/wax (Figure 7c). In the case of pressed PETN/wax, the baseline intensity of the spectrum through Black Sea water was higher than that of distilled water (as expected) and significantly less intense than that of the 10% silica dispersion (Figure 7d).

#### 2.2.2. The Baltic Sea

**Figure 8:**
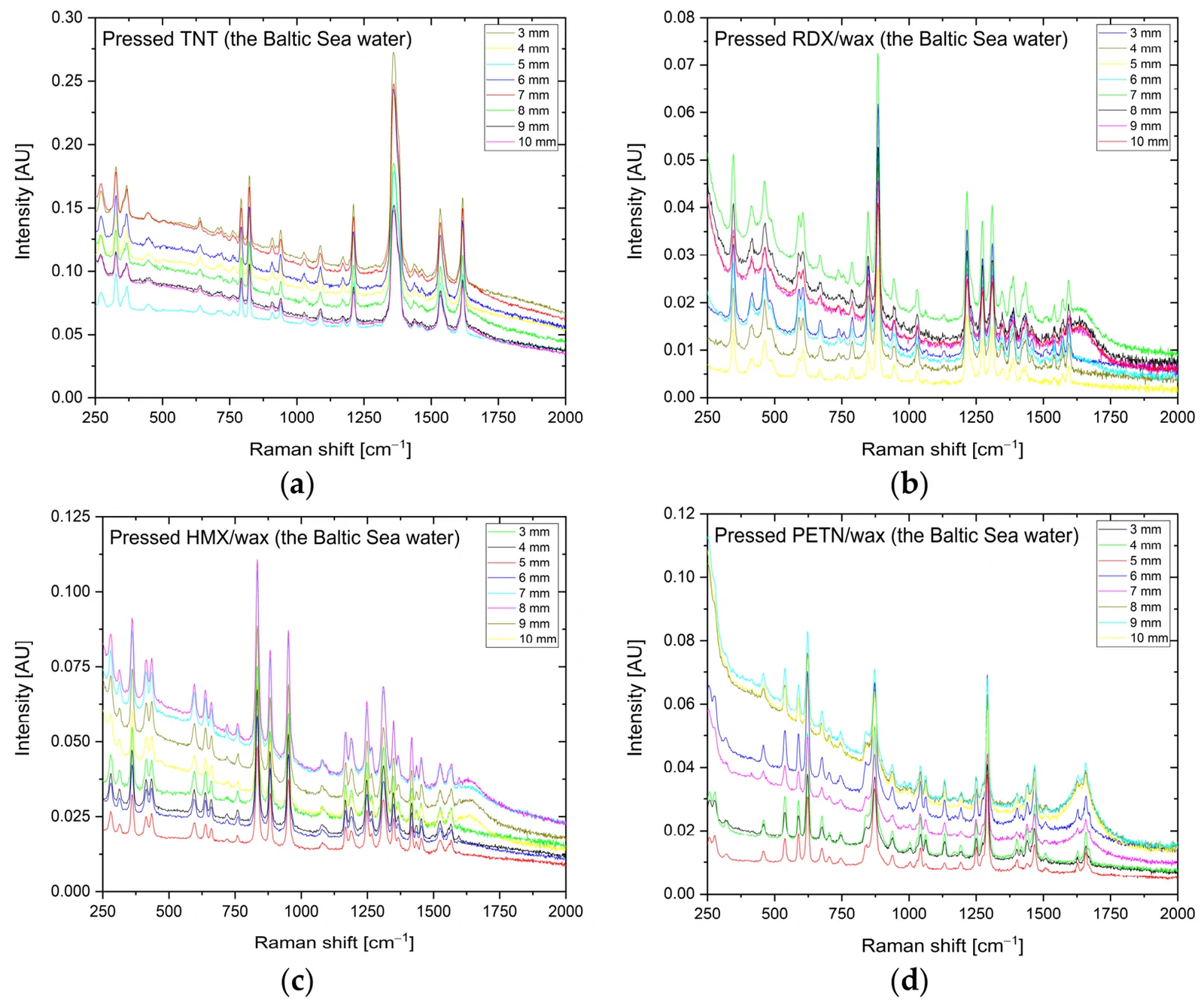
The Raman spectra of pressed charges of explosives through Baltic Sea water: (**a**) the Raman spectra of pressed TNT through Baltic Sea water, (**b**) the Raman spectra of pressed RDX/wax through Baltic Sea water, (**c**) the Raman spectra of pressed HMX/wax through the Baltic Sea water, (**d**) the Raman spectra of pressed PETN/wax through Baltic Sea water.

The influence of Baltic Sea water on the Raman spectrum of the selected high explosives is presented in Figure 8a–d. The spectrum with the least intense baseline was registered at a 5 mm distance, yet the characteristic bands are clearly distinguishable in all cases. Gemini was able to successfully identify all high explosives at all distances.

**Figure 9:**
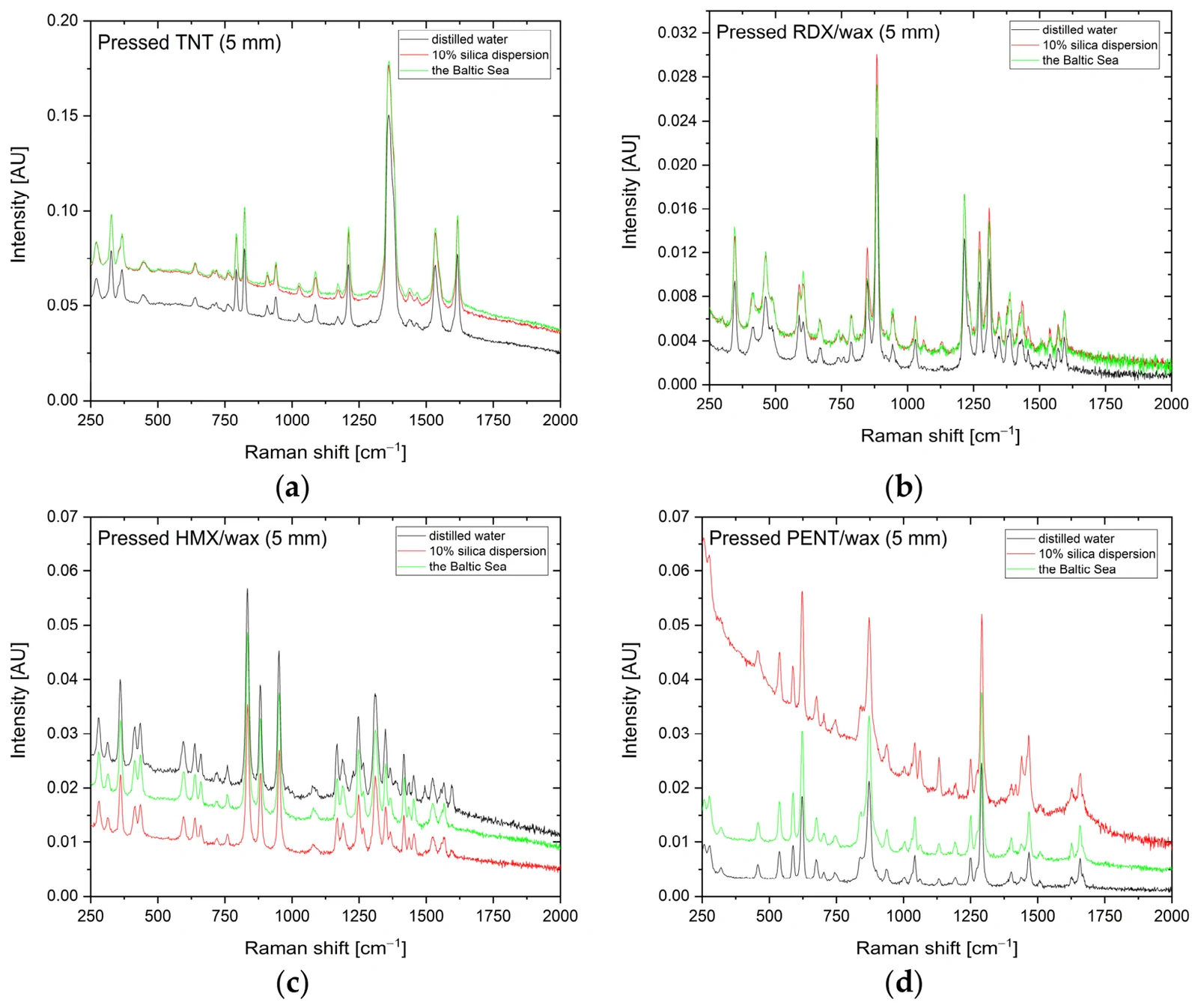
Comparison of the Raman spectra of pressed charges of explosives through Baltic Sea water, distilled water and 10% silica dispersion at 5 mm distance for: (**a**) TNT, (**b**) RDX/wax, (**c**) HMX/wax, (**d**) PETN/wax.

The spectra with Baltic Sea water are compared with distilled water and silica dispersion at 5 mm distance in Figure 9a–d. In the case of pressed TNT, the spectra through Baltic Sea water and 10% silica dispersion are almost identical (Figure 9a). The same goes for pressed RDX/wax (Figure 9b). In the case of pressed HMX/wax, the baseline intensity of the spectrum through Baltic Sea water was higher than that of the 10% silica dispersion (Figure 9c). In the case of pressed PETN/wax, the baseline intensity of the spectrum through Baltic Sea water was higher than that of distilled water and significantly less intense than that of the 10% silica dispersion (Figure 9d).

Selected data from Figure 2, Figure 3, Figure 4, Figure 5, Figure 6, Figure 7, Figure 8 and Figure 9, considering Raman shifts and intensities of characteristic bands, are presented in Table 1, Table 2, Table 3 and Table 4.

## 3. Discussion

During the investigation, four different explosives were tested at distances ranging from 3 to 10 mm in 1 mm increments, using six different types of media. Gemini successfully identified each explosive in every case. The best results were obtained at a distance of 5 mm, corresponding to the focal point of the device; however, no significant decline in spectral quality was observed even at 10 mm—twice the focal length. The different media had no substantial adverse effect on the spectra and, in fact, produced a slight improvement relative to spectra collected through air.

From a chemical perspective, compounds in which the nitro group is bonded to a carbon atom (TNT), an oxygen atom (PETN), or a nitrogen atom (RDX, HMX) were examined. Formally, PETN is a nitroester, so the functional group is a nitrate rather than a nitro group. In RDX and HMX, the nitro group is attached to a nitrogen atom within a ring of alternating carbon and nitrogen atoms. The N–NO_2_ moiety undergoes many of the typical reactions of nitro groups, including stepwise reduction first to a nitroso and ultimately to an amino group. The resulting asymmetrically positioned hydrazine system is unstable and decomposes into simple compounds such as ammonia, nitrogen, or nitrogen oxides. Because the nitrogen atom in the nitro group is in its highest oxidation state, the group is relatively resistant to oxidizing agents.

In TNT the nitro groups display two distinct, high-intensity vibrational stretching modes that are readily observed by Raman spectroscopy: 1354–1362 cm^−1^ (symmetric NO_2_ stretching) and 1549–1624 cm^−1^ (asymmetric NO_2_ stretching) [17]. The observed bands matched the theoretical values. However, their intensity depended on the medium in the optical path.

The solubility of silica in water is very low (below 20 mg L^−1^), so Raman signals arising from silica are of very low intensity and are effectively masked by the much stronger signals of the explosive. In the laser-excited sample, the concentration of energetic molecules is essentially 100%. The closer the sample is to the detector, the smaller the amount of silica present in the optical path.

TNT and RDX are stable compounds and are not expected to have undergone significant degradation since they were dumped at sea during the Second World War; HMX and PETN exhibit comparable stability. Nevertheless, spectra recorded from materials that have remained underwater for decades may still differ from those of laboratory-prepared samples. PETN was not widely employed as a main filling in munitions during the Second World War, serving primarily as a bursting charge, while large-scale production of HMX began only after the war. Today, however, both substances are used in numerous weapons deployed in the conflict between Ukraine and Russia, once again exposing the Black Sea to the risk of contamination.

## 4. Materials and Methods

Pressed charges of four high explosives supplied by “NITRO-CHEM” S.A. (PLC) (Bydgoszcz, Poland) have been used in this research: TNT, RDX (coated with 5% of stearic acid as a phlegmatizer), PETN (coated with 25% of paraffin wax as a phlegmatizer) and HMX (coated with 5% of wax as a phlegmatizer). Each charge was of cylindrical shape and had a diameter of 16 mm and a height of approximately 5 mm. The tap water used in tests was retrieved in Warsaw in February 2025; the water supplier was Warsaw Water Supply Company (Warsaw, Poland). The 40% silica suspension in water used in tests was LUDOX^®^ AS-40 colloidal silica from Sigma-Aldrich (Burlington, MA, USA, product number: 42040). The environmental water samples were collected from the bottom of the Black Sea (Northwestern Romania Coast, depth 15 m) and the Baltic Sea (Słupsk Bank, Słupsk, Poland, depth 40 m).

The analyses were performed using a Gemini™ device (software version number 1.10.0.7726) from ThermoFisher Scientific (Waltham, MA, USA). The Raman mode in Gemini uses a 785 nm laser that was set to high power (250 mW) for measurements. The spectral range of the obtained spectrographs for the Gemini™ device is 250 cm^−1^ to 2875 cm^−1^ and the spectral resolution is 7 cm^−1^ to 10.5 cm^−1^. Each spectrum was collected three times and then averaged.

**Figure 10:**
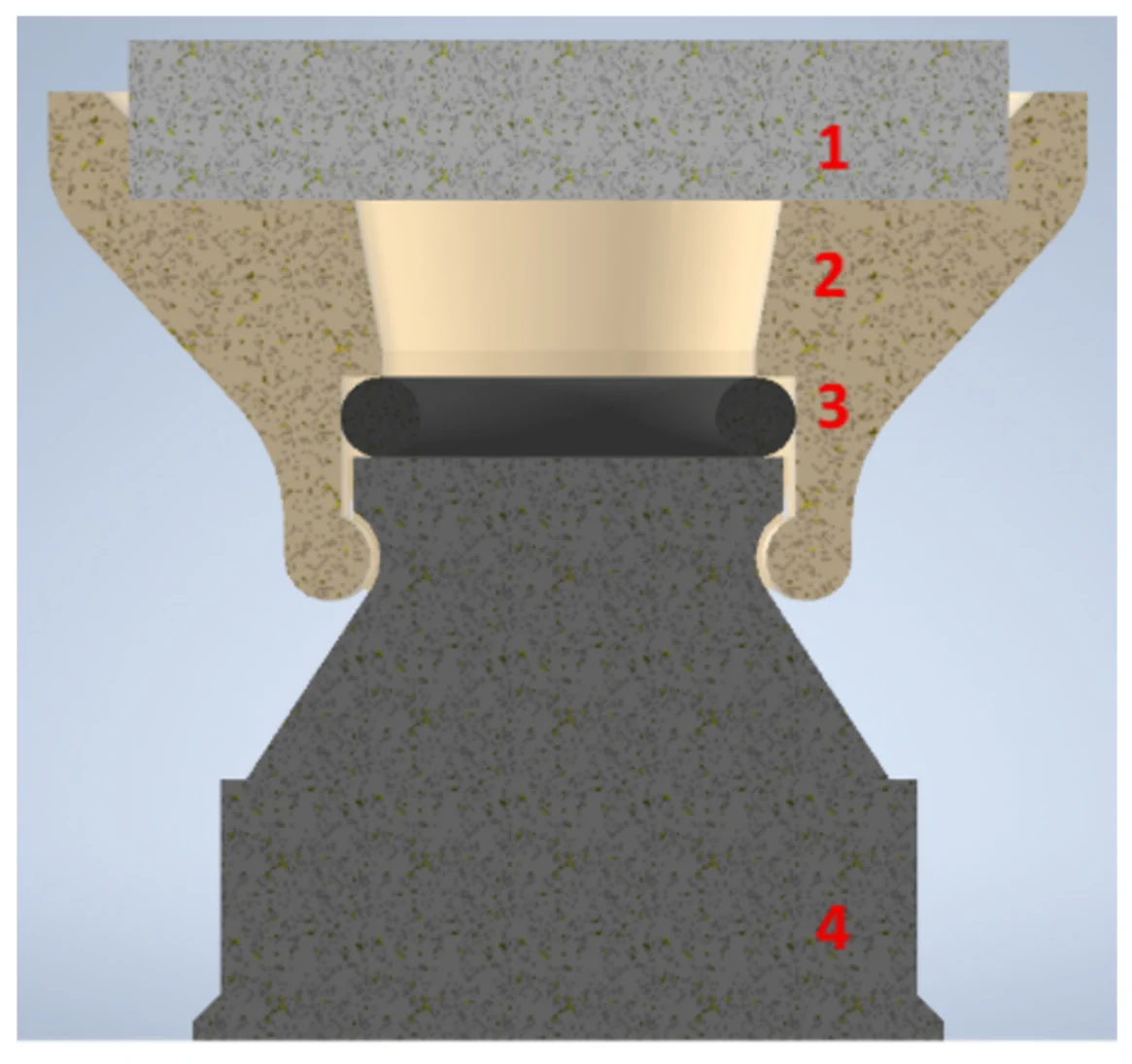
The cross-section of the measurement setup; 1—sample, 2—3D-printed adapter, 3—rubber O-ring, 4—Raman probe.

In order to ensure a fixed distance between the probe and the sample and that no air bubbles were trapped, specially designed adapters (Figure 10) were 3D-printed using a Zortrax Inkspire resin 3D printer (Olsztyn, Poland). A set of 8 adapters with a distance ranging from 3 to 10 mm were prepared. Before the measurement, the adapters were filled with the selected medium and the sample was placed at the top. In order to ensure that the setup was watertight, a rubber O-ring was used.

## 5. Conclusions

Considering all the results, the following conclusions can be drawn:

- Analysis performed through tap water (distance 3–10 mm) had no significant influence on the spectra in comparison to distilled water.
- In most cases the presence of water positively affected the spectra by lowering the baseline intensity in comparison to air.
- Silica dispersions with silica contents ranging from 5 to 40% had a small or moderate influence on the spectra and did not disrupt the identification process in all cases. This phenomenon might be explained by silica’s 785 nm wavelength transparency. The relationship between the silica content and the baseline intensity was not determined due to inconclusive results.
- Black Sea water and Baltic Sea water influenced the spectra slightly by heightening the baseline intensity in the case of pressed TNT, pressed RDX/wax and pressed PETN/wax.
- The Raman spectrometer (Thermo Sci. Gemini) correctly identified each explosive at all distances and with every medium that was used.
- Raman spectroscopy enables non-contact, remote and underwater detection of explosives. The detection and analysis technique can be implemented in Remote Underwater Vehicles (ROVs).

## Figures and Tables

**Table 1:** Characteristic bands of TNT in different media, 5 mm distance.

Medium		TNT Bands					
		C-H Out-of-Plane Bend.	-NO_2_ Def.	C_6_-C Def.	-NO_2_ Sym. Str.	-NO_2_ Asym. Str.	C=C Str.
Distilled water	Shift [cm^−1^]	792	822	1211	1360	1534	1616
	Intensity [AU]	0.069	0.080	0.072	0.151	0.071	0.077
Tap water	Shift [cm^−1^]	792	822	1211	1361	1535	1617
	Intensity [AU]	0.080	0.092	0.083	0.170	0.082	0.089
Air	Shift [cm^−1^]	792	822	1211	1359	1533	1616
	Intensity [AU]	0.102	0.117	0.101	0.197	0.100	0.108
10% silica dispersion	Shift [cm^−1^]	792	824	1211	1360	1533	1618
	Intensity [AU]	0.086	0.100	0.089	0.177	0.088	0.095
40% silica dispersion	Shift [cm^−1^]	792	822	1211	1360	1533	1616
	Intensity [AU]	0.104	0.121	0.107	0.211	0.106	0.116
Black Sea	Shift [cm^−1^]	793	823	1212	1359	1534	1617
	Intensity [AU]	0.076	0.090	0.079	0.161	0.080	0.086
Baltic Sea	Shift [cm^−1^]	792	825	1212	1362	1535	1618
	Intensity [AU]	0.088	0.102	0.091	0.179	0.091	0.098

**Table 2:** Characteristic bands of RDX in different media, 5 mm distance.

Medium		RDX Bands					
		-NO_2_ Wag.	NC_2_ Ring Str.			-NO_2_ Sym. Str.	
Distilled water	Shift [cm^−1^]	589	848	883	1216	1272	1309
	Intensity [AU]	0.006	0.009	0.023	0.013	0.009	0.011
Tap water	Shift [cm^−1^]	592	848	884	1216	1272	1310
	Intensity [AU]	0.009	0.014	0.030	0.018	0.013	0.014
Air	Shift [cm^−1^]	592	849	884	1215	1274	1310
	Intensity [AU]	0.017	0.021	0.049	0.028	0.024	0.027
10% silica dispersion	Shift [cm^−1^]	589	848	883	1216	1273	1309
	Intensity [AU]	0.009	0.012	0.030	0.017	0.014	0.016
40% silica dispersion	Shift [cm^−1^]	590	848	884	1217	1272	1310
	Intensity [AU]	0.023	0.028	0.055	0.031	0.028	0.029
Black Sea	Shift [cm^−1^]	590	848	884	1217	1272	1310
	Intensity [AU]	0.008	0.011	0.027	0.016	0.012	0.014
Baltic Sea	Shift [cm^−1^]	589	846	884	1214	1272	1310
	Intensity [AU]	0.008	0.010	0.027	0.017	0.012	0.015

**Table 3:** Characteristic bands of HMX in different media, 5 mm distance.

**Medium**		**HMX Bands**				
		**-NO_2_ Wag.**	**NC_2_ Ring Str.**			
Distilled water	Shift [cm^−1^]	596	834	881	1168	1188
	Intensity [AU]	0.029	0.057	0.039	0.028	0.025
Tap water	Shift [cm^−1^]	594	834	882	1167	1188
	Intensity [AU]	0.017	0.041	0.027	0.016	0.015
Air	Shift [cm^−1^]	596	835	882	1167	1188
	Intensity [AU]	0.019	0.042	0.028	0.018	0.017
10% silica dispersion	Shift [cm^−1^]	596	834	884	1170	1190
	Intensity [AU]	0.015	0.035	0.023	0.014	0.014
40% silica dispersion	Shift [cm^−1^]	598	834	882	1167	1188
	Intensity [AU]	0.027	0.057	0.039	0.025	0.023
Black Sea	Shift [cm^−1^]	597	835	882	1169	1190
	Intensity [AU]	0.013	0.032	0.021	0.013	0.012
Baltic Sea	Shift [cm^−1^]	598	834	882	1169	1188
	Intensity [AU]	0.023	0.049	0.033	0.022	0.020
**Medium**		**HMX Bands**				
		**N-N Str.**	**-NO_2_ Sym. Str.**			
Distilled water	Shift [cm^−1^]	951	1247		1309	
	Intensity [AU]	0.045	0.033		0.037	
Tap water	Shift [cm^−1^]	952	1248		13,010	
	Intensity [AU]	0.031	0.021		0.025	
Air	Shift [cm^−1^]	952	1246		1310	
	Intensity [AU]	0.032	0.023		0.026	
10% silica dispersion	Shift [cm^−1^]	952	1248		1310	
	Intensity [AU]	0.027	0.019		0.022	
40% silica dispersion	Shift [cm^−1^]	952	1248		1310	
	Intensity [AU]	0.044	0.031		0.036	
Black Sea	Shift [cm^−1^]	952	1248		1310	
	Intensity [AU]	0.024	0.017		0.020	
Baltic Sea	Shift [cm^−1^]	952	1248		1312	
	Intensity [AU]	0.037	0.027		0.031	

**Table 4:** Characteristic bands of PETN in different media, 5 mm distance.

Medium		PETN Bands		
		-NO_2_ In-Plane Def.	N-O Str.	-NO_2_ Sym. Str.
Distilled water	Shift [cm^−1^]	621	872	1291
	Intensity [AU]	0.018	0.021	0.025
Tap water	Shift [cm^−1^]	624	872	1293
	Intensity [AU]	0.026	0.031	0.046
Air	Shift [cm^−1^]	622	872	1291
	Intensity [AU]	0.060	0.060	0.070
10% silica dispersion	Shift [cm^−1^]	623	872	1292
	Intensity [AU]	0.056	0.051	0.052
40% silica dispersion	Shift [cm^−1^]	622	870	1293
	Intensity [AU]	0.034	0.034	0.040
Black Sea	Shift [cm^−1^]	622	872	1291
	Intensity [AU]	0.025	0.027	0.029
Baltic Sea	Shift [cm^−1^]	624	872	1291
	Intensity [AU]	0.031	0.033	0.038

## Data Availability

The raw data supporting the conclusions of this article will be made available by the authors on request.

## Abbreviations

The following abbreviations are used in this manuscript:

TNT	2,4,6-trinitrotoluene
RDX	1,3,5-trinitro-1,3,5-triazacyclohexane
HMX	1,3,5,7-tetranitro-1,3,5,7-tetrazocane
PETN	2,2-Bis[(nitrooxy)methyl]propane-1,3-diyl dinitrate, pentaerythritol tetranitrate
